# A cross-sectional survey to establish *Theileria parva* prevalence and vector control at the wildlife-livestock interface, Northern Tanzania

**DOI:** 10.1016/j.prevetmed.2021.105491

**Published:** 2021-11

**Authors:** Fiona K. Allan, Emmanuel Sindoya, Katherine E. Adam, Mechtilda Byamungu, Rachel S. Lea, Jennifer S. Lord, Geofrey Mbata, Edith Paxton, Furaha Mramba, Stephen J. Torr, W. Ivan Morrison, Ian Handel, Liam J. Morrison, Harriet K. Auty

**Affiliations:** a Roslin Institute, Royal (Dick) School of Veterinary Studies, University of Edinburgh, Midlothian, EH25 9RG, United Kingdom; b Minstry of Livestock and Fisheries, Serengeti District Livestock Office, Mugumu, Tanzania; c Innogen Institute, Science Technology and Innovation Studies; School of Social and Political Science, University of Edinburgh, Old Surgeons’ Hall, High School Yards, Edinburgh, United Kingdom; d Tanzania Veterinary Laboratory Agency, Dar es Salaam, Tanzania; e Department of Vector Biology, Liverpool School of Tropical Medicine, Liverpool, United Kingdom; f Vector and Vector-borne Diseases Research Institute, Tanga, Tanzania; g Institute of Biodiversity, Animal Health & Comparative Medicine, College of Medical, Veterinary & Life Sciences, University of Glasgow, United Kingdom (Previously Epidemiology Research Unit, SRUC, Inverness, United Kingdom)

**Keywords:** *Theileria parva*, *Rhipicephalus appendiculatus*, East Coast fever, Vector, Acaricide, Wildlife-livestock interface

## Abstract

East Coast fever (ECF) in cattle is caused by the protozoan parasite *Theileria parva*, transmitted by *Rhipicephalus appendiculatus* ticks. In cattle ECF is often fatal, causing annual losses >$500 million across its range. The African buffalo (*Syncerus caffer*) is the natural host for *T. parva* but the transmission dynamics between wild hosts and livestock are poorly understood. This study aimed to determine the prevalence of *T. parva* in cattle, in a 30 km zone adjacent to the Serengeti National Park, Tanzania where livestock and buffalo co-exist, and to ascertain how livestock keepers controlled ECF and other vector-borne diseases of cattle.

A randomised cross-sectional cattle survey and questionnaire of vector control practices were conducted. Blood samples were collected from 770 cattle from 48 herds and analysed by PCR to establish *T. parva* prevalence. Half body tick counts were recorded on every animal. Farmers were interviewed (n = 120; including the blood sampled herds) using a standardised questionnaire to obtain data on vector control practices. Local workshops were held to discuss findings and validate results.

Overall prevalence of *T. parva* in cattle was 5.07% (CI: 3.70−7.00%), with significantly higher prevalence in older animals. Although all farmers reported seeing ticks on their cattle, tick counts were very low with 78% cattle having none. Questionnaire analysis indicated significant acaricide use with 79% and 41% of farmers reporting spraying or dipping with cypermethrin-based insecticides, respectively. Some farmers reported very frequent spraying, as often as every four days. However, doses per animal were often insufficient.

These data indicate high levels of acaricide use, which may be responsible for the low observed tick burdens and low ECF prevalence. This vector control is farmer-led and aimed at both tick- and tsetse-borne diseases of livestock. The levels of acaricide use raise concerns regarding sustainability; resistance development is a risk, particularly in ticks. Integrating vaccination as part of this community-based disease control may alleviate acaricide dependence, but increased understanding of the *Theileria* strains circulating in wildlife-livestock interface areas is required to establish the potential benefits of vaccination.

## 1 Introduction

East Coast fever (ECF) in cattle, caused by the tick-borne protozoan parasite *Theileria parva,* occurs throughout a large region of eastern, central and southern Africa (Norval et al., 1992). ECF is a major cause of death in cattle in affected areas, resulting in up to 70% mortality in susceptible breeds of cattle (Lynen et al., 2005; Homewood et al., 2006). Global economic losses due to ECF have been estimated to be US $596 million annually (GALVmed, 2019). Hence, the disease is a major constraint to the development of the livestock sector throughout affected areas (Chenyambuga et al., 2010).

Infection with *T. parva* is endemic in most areas of East Africa infested by the tick vector (Coetzer and Tustin, 2004), but mortality in infected animals varies, depending largely on the breed of cattle and the extent to which control measures are applied (Norval et al., 1992). Unlike other important tick borne pathogens such as *Babesia* and *Anaplasma,* which cause severe disease in adult cattle but mild clinical symptoms in calves allowing the establishment of a state of endemic stability in affected areas (Jonsson et al., 2012), there is no clear evidence of age-associated resistance to disease with *T. parva*. Therefore, calves can suffer severe fatal disease. However, unlike European *Bos taurus* breeds of cattle, which are highly susceptible to *T. parva*, indigenous East African zebu cattle (*Bos indicus*) residing in tick-infested areas exhibit a degree of resistance to the disease (Laisser et al., 2017) - infected calves generally suffering less than 10% mortality in the absence of any disease control measures (de Clare Bronsvoort et al., 2013; Kiara et al., 2014). However, experimental studies have shown that this resistance is not absolute, but is dependent on the number of infected ticks with which the animals are challenged (Barnett, 1957; Ndungu et al., 2005). Animals that recover from infection in the field develop immunity to the parasite, which is boosted by further parasite challenge (Gachohi et al., 2012). Consequently, in endemic settings where there is constant exposure to *T. parva*, ECF is rarely observed in adult indigenous zebu cattle, since most animals develop immunity following exposure as calves (Jonsson et al., 2012). In contrast, if challenge by ticks is only sporadic or some tick control measures are applied, clinical disease may occur across all age groups (Gachohi et al., 2012).

After infection, a low level parasitaemia persists in cattle, referred to as the carrier state (Young et al., 1986). Although parasites are generally not detected microscopically in carrier animals, their presence can be demonstrated by PCR (Skilton et al., 2002) and they represent a source of infection for feeding ticks (Kariuki et al., 1995). The prevalence of infection in such ticks is low and they exhibit low levels of infection in their salivary glands (Norval et al., 1992). Nevertheless, mathematical modelling studies have indicated that in endemic areas, infection of ticks is sustained predominantly by feeding on carrier animals (Medley et al., 1993).

The African buffalo (*Syncerus caffer*) is considered the primary host for *T. parva,* but does not suffer disease. Buffalo living in endemic areas are essentially all infected with *T. parva* (Young et al., 1978). When buffalo-derived *T. parva* is transmitted to cattle, they rapidly develop clinical disease (often referred to as Corridor disease), but in most cases the parasites do not differentiate to piroplasms in cattle and therefore are not transmissible by ticks (Schreuder et al., 1977; Mbizeni et al., 2013; Latif et al., 2019). Therefore, infection with most buffalo-derived parasites is not maintained in the cattle population (Pelle et al., 2011).

Vaccination is available for ECF control, using a *T. parva* infection and treatment method (ITM) (Radley et al., 1975b, 1975c), but although this has significant uptake in some ECF-affected areas (Lynen, 2005; Di Giulio et al., 2009; Kazungu et al., 2015b), its widespread use is limited by a requirement for a coldchain for storage and distribution of the vaccine. Moreover, there is evidence that it is not effective in areas with heavy challenge with buffalo *T. parva* (Radley et al., 1979; Radley, 1981; Bishop et al., 2015; Sitt et al., 2015). One explanation for this is that the ITM vaccine does not provide sufficient protection against the much greater genetic and antigenic diversity observed in buffalo-derived *T. parva* (Conrad et al., 1989; Oura et al., 2011; Pelle et al., 2011; Hemmink et al., 2018; Kerario et al., 2019; Allan et al., 2021). East Coast fever is therefore most commonly controlled by targeting the tick vector through the application of acaricides (Kivaria, 2006), which are frequently used by smallholder farmers in affected areas (Chenyambuga et al., 2010; Kazungu et al., 2015a; Kerario et al., 2017b). However, acaricides need to be applied frequently to be effective and some farmers have been reported to underdose the acaricides to reduce costs (Laisser et al., 2014). Application of acaricides, as well as reducing infection with *T. parva*, also results in many animals remaining susceptible to infection, which can result in disease outbreaks if the supply of acaricides is interrupted or they are applied inappropriately (Jongejan and Uilenberg, 2004). Hence, given ECF epidemiology is affected by variation in tick challenge, breed of cattle and the type and efficacy of vector control used, effective application of control measures requires knowledge of these factors in the target area.

Although ECF is often cited as one of the most important disease concerns in wildlife-livestock interface areas (Nthiwa et al., 2019), there are limited data describing the epidemiology of ECF in areas where cattle and buffalo co-exist. The Serengeti-Mara Ecosystem was identified as a key area where cattle surrounding the protected areas would be at risk of *T. parva* infection from buffalo, based on the distribution of cattle, buffalo, *R. appendiculatus* and *T. parva* (Wint and Kiara, 2017). However, the epidemiology of ECF in this area has not been studied extensively. During the dry season, cattle are often grazed within the protected areas (Laisser et al., 2014). The presence of buffalo within and around the boundaries of the unfenced national park, along with *R. appendiculatus*, provides the potential for cattle to become infected with both cattle- and buffalo-derived *T. parva* while grazing. This system, therefore, provides an opportunity to improve understanding of epidemiology of ECF in a wildlife-livestock interface area and identify how to best optimise control. This study used a mixed methods approach to (i) measure the prevalence of *T. parva* in cattle around the protected area (ii) assess control measures currently implemented by livestock keepers, and (iii) identify the barriers to improved control.

## 2 Materials and methods

### 2.1 Ethical approval and permits

Ethical approval was gained from Scotland’s Rural College (SRUC) Animal Experimentation Committee, the Tanzania Wildlife Research Institute (TAWIRI) and Commission for Science and Technology (COSTECH) (Research Permit Number 2016-32-NA-2016-19). The questionnaire, participant consent form and participant information sheet were all approved by the Royal (Dick) School of Veterinary Studies (R(D)SVS), University of Edinburgh, Human Ethical Review Committee (HERC_00_16).

### 2.2 Study area

**Fig. 1:**
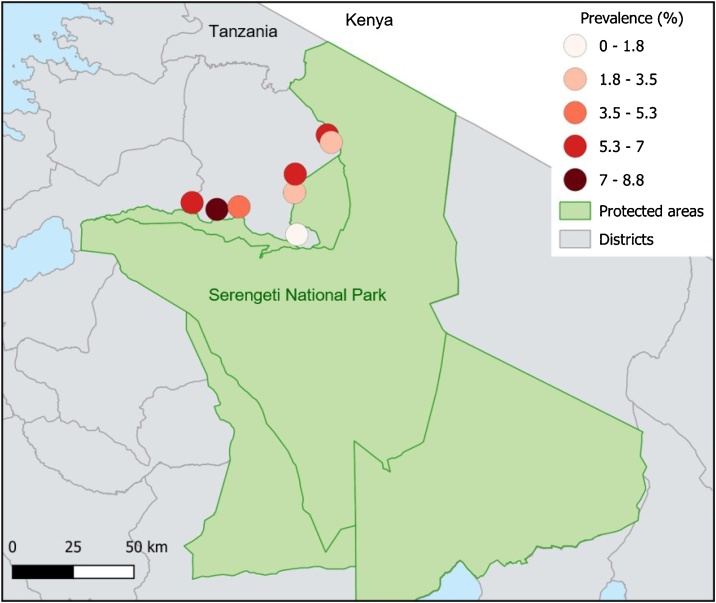
Location of sampled villages and *T. parva* prevalence. Villages shown in shades of red, representative of *T. parva* prevalence, as detailed in the legend. Villages were selected from those close to the protected area boundaries in Serengeti District. Protected wildlife areas are shown in green and comprise Serengeti National Park, Ngorongoro Conservation Area and Grumeti, Ikorongo and Maswa Game Reserves.

The study was carried out in Serengeti District, at the north western border of the Serengeti National Park, Tanzania (Fig. 1) between July 2016 and February 2017. Communities here practice livestock keeping as well as mixed crop farming (Estes et al., 2012). Cattle in this area mostly comprise indigenous short horn zebu or Sahiwal, Boran or Mpwapwa zebu cross breeds; livestock density is high (TAWIRI, 2016). Cattle are managed extensively and grazed in common grazing areas or other local areas. Cattle grazing is not permitted in protected areas, but is reported to occur (Laisser et al., 2014). In the most recent aerial census carried out (TAWIRI, 2016), a total of 1,210,846 ± 19,679 cattle were counted in the Serengeti ecosystem; in Serengeti District, cattle density is estimated at 30 cattle/km^2^ (Robinson et al., 2014). The area is approx. 1410 m above sea level and has a tropical climate, with a mean monthly maximum temperature of 27−28 °C. The hotter months of October to March have a minimum temperature of 16 °C and the cooler months of May to August a minimum of 13 °C. Typical rainfall pattern is bimodal; long rains are from March until May and short rains from November to December (Sinclair and Arcese, 1995). Vegetation in the protected areas of Serengeti ecosystem comprises savannah grasslands and acacia woodlands (Boone et al., 2006) whilst areas outside the protected area have predominantly been converted to agriculture (Estes et al., 2012).

### 2.3 Cattle survey - study design

A multistage stratified sampling strategy was used to select herds (Wesonga et al., 2015). Out of 78 villages in Serengeti District, 18 are located adjacent to the protected area boundary (defined here as village centre within 5 km of boundary). Out of these, eight villages were randomly selected. Within each village two sub-villages were selected at random: sub-villages are administrative village divisions, with usually two to six sub-villages per village. Sub-village authorities provided a list of livestock-owning households, and three herds were selected randomly from each sub-village. Overall, 48 herds were selected. In accordance with the sample size calculation, a maximum of 20 cattle per herd were sampled, or all cattle if the herd size was fewer than 20. Cattle younger than 6 months were not included as farmers usually practice zero-grazing in order to protect them from disease (Swai et al., 2009; Muhanguzi et al., 2014). The resulting sample size was 770, sufficient for establishing a prevalence of *T. parva* of 20.0% (Swai et al., 2007) at 95% confidence and precision of 5.0%, and taking into account clustering within herds with a design effect of 2.1, assuming a mean of 15 animals sampled per herd (intra-cluster correlation coefficient estimated at 0.08 based on previous data). Written informed consent was gained at each household; all selected households chose to participate.

Cattle were selected randomly for sampling as far as possible. A 10 mL blood sample was collected from the jugular vein into a PAXgene tube (PAXgene Blood DNA System, Qiagen). Cow-level data were collected for each animal sampled, including age, sex, origin (homebred or bought-in), health status at time of sampling (sick or healthy), body condition score and any treatments given in the last six months. Age was defined according to dentition (Turton, 2001). Body condition score (BCS) was recorded numerically from 1 to 5, where 1 = very poor and 5 = very good, and was then coded into categories ‘poor’ (<2.5), ‘fair’ (2.5–2.7), or ‘good’ (>2.7) for analysis. Ticks were quantified on individual animals by examining the upper exposed half of the body, based on guidelines described by Walker et al. (2003). In order to minimise the period of time the animal was restrained, a tick count scoring system was created, similar to that described by Simuunza et al. (2011) where score 0 represents 0 ticks, score 1 is 1−10 ticks, score 2 is 11–50 ticks and score 3 is more than 50. A count was carried out for the total half body as well as a separate count for the ears only, the predilection site for *R. appendiculatus*.

### 2.4 *T. parva* detection

The PAXgene Blood DNA Kit (Qiagen) was used to isolate genomic DNA. DNA was stored at −20 °C or −80 °C until further analysis. All 770 cattle samples were screened for *T. parva* using a nested PCR (nPCR) targeting the *T. parva-*specific p104 gene, as previously described (Skilton et al., 2002; Odongo et al., 2009). This nPCR assay is estimated to detect 0.4 parasites/μL (equating to a blood parasitaemia of 9.2 × 10^−6^%) (Odongo et al., 2010) with a reported specificity of 100% (Skilton et al., 2002), thus making it suitable for detecting the expected low levels of *T. parva* infections in field samples. PCR mix consisted of 12.5 μL Quick-Load Taq 2X Master Mix (New England Biolabs), 1 μL of each primer (10μM), 10 μL nuclease-free water and 1 μL of DNA template (total 25 μL reaction). For the second round template the first round product was diluted 1:100 in dH_2_O. The nPCR reactions were carried out in a thermal cycler (MJ Research PTC-200 Engine) and the nPCR products were visualised by UV trans-illumination in a 1.5% agarose gel containing GelRed (Biotium) after electrophoresis.

### 2.5 Questionnaire survey – study design

A structured questionnaire survey (Supplementary File) was designed to gather specific information on vector control practices. The questionnaire was conducted verbally in Kiswahili by an experienced enumerator. Participants were given an information hand-out in Kiswahili which was explained verbally, and written consent was obtained. The questionnaire included both closed and open-ended questions and covered farmer demographic information and herd management, farmer knowledge of vectors, vector control methods, and knowledge of East Coast fever. Farmers were shown five different insect vector images and asked to identify a tick. Sample size was calculated for questionnaire distribution and was carried out for a yes/no question based on an estimated proportion of 50%, desired precision of 10% and 95% confidence intervals, resulting in a minimum sample size of 97. The questionnaire was administered to each of the 48 herds where cattle sampling was conducted, and an additional 49 herds were randomly selected from the same subvillages for questionnaire administration only (referred to as ‘cross-sectional herds’ hereafter). In addition, 23 farmers already involved in a separate longitudinal study in the same area completed the questionnaire (hereafter referred to as ‘longitudinal herds’). These farmers had volunteered to participate in the study and were likely to be more proactively concerned with health interventions.

### 2.6 Workshops – study design

Two workshops were held in February 2017 with participants including livestock extension officers, participants from the surveys and village leaders from the same villages. The same workshop was conducted twice to allow sufficient people to participate. Each workshop lasted one day and included small group discussion, with each group led by a Kiswahili-speaking facilitator, and plenary discussion. Topics covered included clinical signs of ECF, perceptions about ECF, reasons for choosing or not choosing different control options and sources of information on ECF and vector control. Data were also collected regarding which villages had a functional dip tank. Preliminary results from the prevalence study were shared with participants.

### 2.7 Data analysis

Data analysis was conducted in Excel, R (R Core Team, 2018) and QGIS version 2.18.28. Data were cleaned using R ‘tidyverse’, including coding of missing data and categorising continuous variables. Prevalence of *T. parva* was established at village-level and herd-level. A random effects model was used to estimate the overall and village level prevalences, adjusting the estimates and confidence intervals for the clustering of animals within villages and subvillages.

Logistic regression models were used to assess cow-level risk factors of being PCR positive for *T. parva*, as well as analysis of herd-level risk factors for vector control. Cow-level variables considered for the model were age, sex, origin, body condition score, half-body tick count and ear ticks. Analysis was performed at the univariable level and variables were considered for inclusion in a multivariable model if significant at p < 0.1. Where generalised linear models estimated large standard errors due to complete separation of outcomes with predictors, Firth’s regression and Fisher’s Exact test were used. Likelihood ratio test (LRT) comparisons were used to assess the significance of overall variables of being PCR positive. Numerical outcomes were modelled using univariable linear regression. Statistical tests were considered significant at p ≤ 0.05.

## 3 Results

### 3.1 Prevalence of *T. parva*

Overall, of the 770 cattle sampled, 39 were positive by p104 PCR for *T. parva* (5.07%, CI: 3.70−7.00%). Prevalence varied from 0.00%–16.67% at herd level. Herd was not found to be a significant risk factor for *T. parva* prevalence (p = 0.274). Prevalence of *T. parva* at village level (Table 1) ranged from 0.00%–8.77% (Fig. 1). Prevalence adjusted for clustering was calculated for all villages except village 8, and ranged from 2.50%−9.60%. The zero prevalence in village 8 differed significantly from that of other villages (p = 0.019) but there was no significant difference in prevalence of *T. parva* between the other villages.

**Table 1:** Prevalence of *T. parva* distribution by village.

Village	Cattle number	Raw prevalence	Adjusted prevalence and 95 % CI
1	113	7/113 (6.19%)	6.60% (3.10−14.20)
2	81	2/81 (2.47%)	2.50% (0.60−10.30)
3	115	6/115 (5.22%)	5.50% (2.40−12.50)
4	91	6/91 (6.59%)	8.10% (3.50−18.60)
5	92	3/92 (3.26%)	3.40% (1.10−10.90)
6	114	10/114 (8.77%)	9.60% (5.00−18.40)
7	77	5/77 (6.49%)	7.20% (2.90−18.00)
8+	88	0/88 (0.00%)	NA

+ Due to zero prevalence and therefore complete separation of the data in village 8, it was not possible to include this village in the generalised linear model and so Fisher’s Exact test was used for this village instead. It was also not possible to include village 8 in the random effects model for adjusted prevalence.

### 3.2 Cow-level risk factor analysis

Distribution of cattle age, sex, origin, body condition score, and tick counts are shown in Table 2. All cattle were reported healthy at the time of sampling. *T. parva* prevalence differed significantly by age, with older cattle (4.5−11 years) having increased risk (OR = 3.47, p = 0.009) of being positive by PCR for *T. parva* compared to the youngest cattle. There was no significant difference in prevalence between the other age categories and the reference level.

**Table 2:** Cow-level factors associated with *T. parva* prevalence based on logistic regression. REF = reference level.

Variable	Factor level	Total	*T. parva* positive (%)	OR	95 % CI	LRT value	p-value
Age						5.5	0.019*
Age category (years)	0.5−1.5	191	7/189 (3.70%)	REF			
	1.5–2.5	164	4/164 (2.44%)	0.65	0.016−2.19		0.498
	2.5−3.0	113	9/113 (7.96%)	2.25	0.81−6.46		0.118
	3.0−4.5	175	5/175 (2.86%)	0.76	0.22−2.44		0.652
	4.5−11	119	14/119 (11.76%)	3.47	1.39−9.39		0.009*
Cattle sex						0.35	0.554
	Male	208	9/208 (4.32%)	REF			
	Female	561	30/559 (5.37%)	1.25	0.61−2.85		0.561
Origin						0.62	0.429
	Bought-in	129	6/129 (4.65%)	REF			
	Homebred	636	33/636 (5.19%)	1.45	0.61−4.29		0.449
Body condition score						1.63	0.202
	Poor	339	16/339 (4.72%)	REF			
	Fair	304	18/304 (5.92%)	1.27	0.63−2.56		0.498
	Good	119	5/119 (4.20%)	0.89	0.28−2.31		0.816
Half body tick count						0.15	0.699
	No ticks (0)	600	32/599 (5.34%)	REF			
	Few ticks (1−10)	160	6/159 (3.77%)	0.69	0.26−1.58		0.423
	Some ticks (11 > 50)	9	1/9 (11.11%)	2.22	0.12−12.61		0.460
Ear ticks†						2.98	0.084
	Present	28	0/28 (0.00%)	REF			
	Absent	738	39/738 (5.28%)	25.1	0.04−67132		1.00

† Due to complete separation of the data, Firth’s regression was used to calculate the OR for Ear ticks and *T. parva* prevalence.

* Significant difference.

### 3.3 Questionnaire descriptive analysis

Analysis was carried out on all questionnaires, cross-sectional and longitudinal combined (n = 120), as well as separately due to their differences in recruitment methods. Results are stated for each group separately when they differed significantly. Most participants were male (77.5%, 93/120) and 65% (78/120) of participants were the head of the household.

#### 3.3.1 Cattle demographics

All participants provided information on the number of cattle they owned. In the longitudinal herds (n = 23), herd size ranged from 46 to 1000 (mean 218.6, median 150) and in the cross-sectional herds (n = 97) numbers ranged from 4 to 280 cattle (mean 42.2, median 25), with a significant difference (p = <0.0001) in the mean herd sizes. Most of the farmers (58.3%, 70/120) owned all of the cattle on their farm, with 41.6% (50/120) having cattle also belonging to someone else. Most farmers (82.5%, 99/120) reported that most of their cattle were homebred; when asking about individual animals, on average 83% of cattle at each cross-sectional household were homebred (range 10%–100%). The majority of farmers also kept sheep (77.5%, 93/120) and goats (74.2%, 89/120).

#### 3.3.2 Cattle movement

All farmers reported keeping their cattle at home overnight, i.e. returning nightly from grazing or watering destinations. Farmers reported travelling between 0−7 km daily for water in the wet season (mean 1.38, median 1) and 0.3−22 km in the dry season (mean 3.47, median 2.5), with similar distances reported for grazing. Approximately one quarter of farmers (24.2%, 29/120) reported sending their cattle away for periods of time, for grazing or watering, as draught or milking animals and for weaning, for periods of time between 0–12 months (mean 2.5, median 2).

#### 3.3.3 Knowledge of vectors

Of the 120 farmers interviewed, 118 (99.3%) were able to identify a tick correctly. All farmers reported seeing ticks on their cattle all year round, with peak times being June until September. Ticks were mostly seen on cattle while at grazing. When asked specifically if they saw ticks on the ears of their cattle 83.8% (98/117) reported they did. When farmers were asked if they knew what diseases ticks transmitted, 23.1% (27/117) thought that ticks transmitted ECF (14.9%, 14/94 cross-sectional; 56.5%, 13/23 longitudinal). Farmers also reported other diseases (or clinical signs) that they associated with ticks, namely Anaplasmosis, Babesiosis, ECF, heartwater, anaemia, enlarged lymph nodes, and high fever.

#### 3.3.4 Vector prevention

Almost all farmers (99.2%, 119/120) reported doing some form of tick prevention and 97.5% (117/120) were using products for tick prevention via dipping or spraying. Two farmers from the longitudinal group reported hand-picking as tick prevention. The majority of farmers (86.7%, 104/120) reported doing tick prevention all year round (83.5%, 81/97 cross-sectional; 100%, 23/23 longitudinal), with only 12.5% (15/120) only doing so when tick numbers were considered high.

**Fig. 2:**
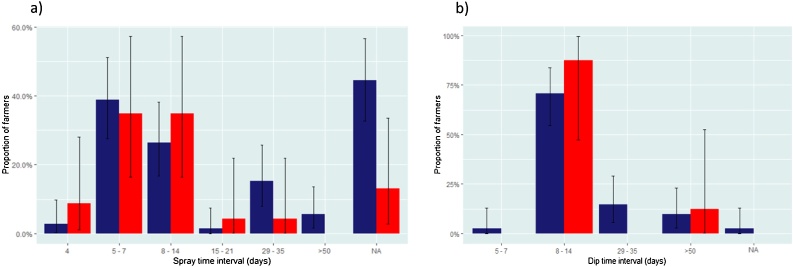
a) Time intervals of acaricide spraying and b) Time intervals of acaricide dipping. Values shown were reported by farmers selected as part of a randomised cross-sectional study (blue) and by farmers participating in a non-randomised longitudinal study (red). N/A = Non-responses. Error bars indicate 95% confidence intervals.

When asked about how they were applying tick prevention products, 79.2% (95/120) of farmers were spraying their cattle (74.2%, 72/97 cross-sectional; 100%, 23/23 longitudinal) and 40.8% (49/120) of farmers were dipping their cattle. Farmers were asked how they applied spray products; 75.8% (91/120) described spraying “all over the body”. The time interval for spraying cattle ranged from every 4–270 days (overall mean 21.2, median 10; cross-sectional 4–270 days, mean 24.44, median 10; longitudinal 4–30 days, mean 10.65, median 7.5; Fig. 2a), with six farmers reporting spraying cattle only when they saw ticks or when tick numbers were considered high rather than at a predetermined interval.

The time interval for dipping cattle ranged from every 7–150 days (overall mean 24.40, median 14 days; cross-sectional 7–150 days, mean 25.33, median 14; longitudinal 14–60 days, mean 19.75, median 14; Fig. 2b).

**Fig. 3:**
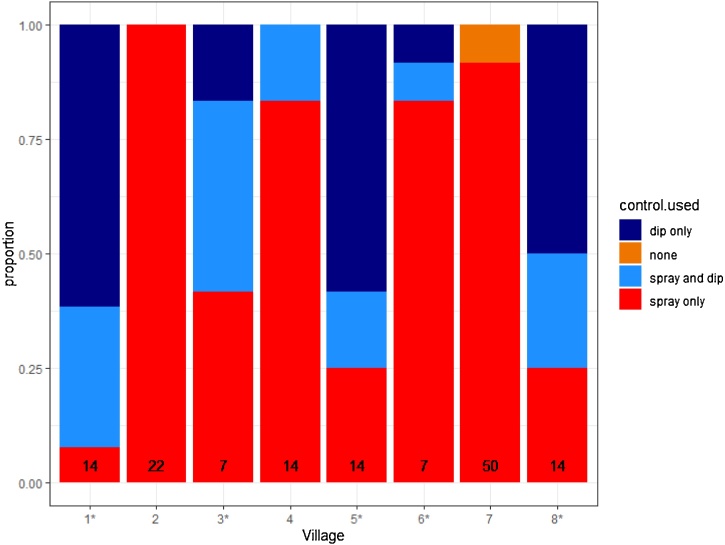
Control methods being used by farmers in each village. The proportions of farmers reporting application of insecticides on their cattle by spraying, using a dip tank, both or neither are shown. Villages that have a dip tank are indicated with an asterisk. Numbers indicate the median frequency of application in days.

Control by village was assessed (Fig. 3). Three of the eight villages did not have functioning dip tanks.

#### 3.3.5 Knowledge of ECF

Almost half the farmers (45.8%, 55/120) had heard of ECF (35.1%, 34/97 cross-sectional; 91.3%, 21/23 longitudinal) and one third (34.7%, 41/118) reported that they knew the clinical signs associated with ECF (23.2%, 22/95 cross-sectional; 82.6%, 19/23 longitudinal). A range of clinical signs considered attributable to ECF were reported; correct signs reported were anaemia, anorexia, diarrhoea, enlarged lymph nodes, laboured breathing, and nasal discharge. Additional signs reported that are not typically associated with ECF were coughing, collapse, circling, drooling, lacrimation, rough hair coat, shivering, and weight loss. Of the farmers who were aware of ECF, 54.5% could identify at least two correct clinical signs (50.0%, 17/34 cross-sectional; 61.9%, 13/21 longitudinal).

Only 29.2% (35/120) farmers knew that ticks caused ECF (20.6%, 20/97 cross-sectional; 65.2%, 15/23 longitudinal). Of the farmers who had heard of ECF, 36.4% (20/55) farmers reported having cases of ECF in their cattle (9.1%, 5/55 were cross-sectional; 27.3%, 15/55 were longitudinal). These farmers each reported having between 0 and 60 cases that they identified as ECF in the past one year (overall mean 6.25, median 1.5; cross-sectional 1–12 cases, mean 1.66, median 0; longitudinal 0–60 cases, mean 10.29, median 4). Of the 20 farmers who had suspected cases, 12 farmers (3 cross-sectional, 9 longitudinal) reported deaths that they attributed to ECF. Of these, the numbers of deaths that each farmer attributed to ECF was 0–12 (overall mean 1.59, median 0; cross-sectional 0–9 deaths, mean 1.2, median 0; longitudinal 0–12 deaths, mean 1.94, median 1).

Farmers were asked if they used prevention methods for ECF. Of the 38 who responded, 32 (84.2%) reported using prevention methods; spraying, dipping or both were the most common control options reported. One cross-sectional farmer used oxytetracycline as a prevention method for ECF. No farmer reported using the ECF vaccine (Muguga Cocktail ITM). When asked why they were not using the vaccine, out of 40 farmers who responded, the reasons stated were that they did not know there was a vaccine (37/40, 18 cross-sectional, 19 longitudinal); they did not know about ECF (1 cross-sectional), or the vaccine was too expensive (1 longitudinal).

All farmers reported using oxytetracycline to treat suspected cases of ECF, ranging from 10 to 30% concentration. Three longitudinal farmers reported using Butalex (buparvaquone) and two reported seeking veterinary advice.

#### 3.3.6 Vector prevention products

**Fig. 4:**
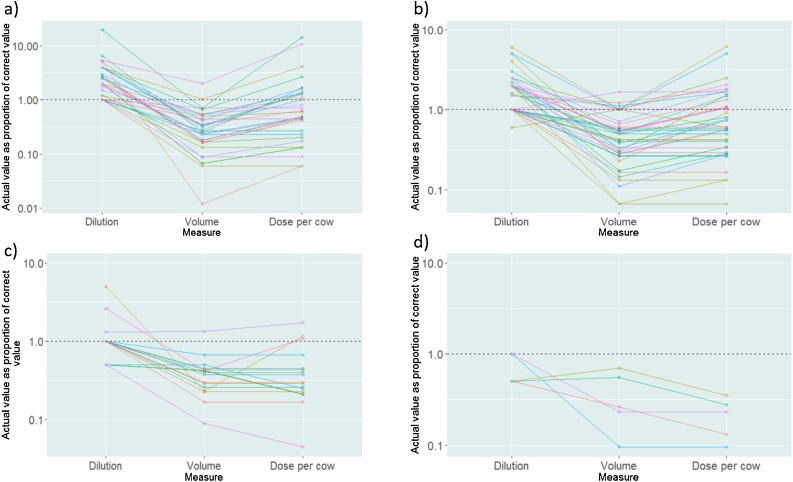
Scatterplots showing dilution, volume used and dose per cow for four acaricide products: a) Albadip; b) Paranex; c) Cybadip; and d) Tantix. The value for each herd is shown as a proportion (log scale) of the correct dose, where values <1.0 (grey dashed line) represent underdosing; different coloured points represent different herds and lines connect individual herds.

Farmers described using four acaricide formulations, namely Albadip (alphacypermethrin 10%), Paranex (alphacypermethrin), Cybadip (cypermethrin 15%) and Tantix (High-cis cypermethrin 10%), with some farmers reporting use of more than one. Farmers were asked how they diluted the product and how much diluted product they applied to each animal. Many farmers made up overly concentrated product, but administered an insufficient volume per animal (Fig. 4). The proportion of farmers giving an adequate amount of active ingredient per animal was 0% for those using Tantix, 18.2% using Cybadip, 41.9% using Paranex and 43.5% for Albadip (Fig. 4). Farmers reported using dilutions of 0.5−1 mL/litre for Tantix, 0.5–2.6 mL/litre for Cybadip, 0.3−3 mL/litre for Paranex, and 0.5−10 mL/litre for Albadip (recommended dilution 1 mL/litre, 1 mL/litre, 0.5 mL/litre, and 0.5 mL/litre, respectively) (Fig. 4).

The same four cypermethrin acaricides were used for dipping. As the dip tanks are community-run, the acaricide products are made up by livestock officers. Farmers paid an arranged price of 100 TZS (equivalent to approximately $0.05) per cow.

Farmers were asked if the acaricides they were using had any other benefits; of the 118 who responded, 61.9% (overall 73/118; cross-sectional 58.9%, 56/95; longitudinal 73.9%, 17/23) believed they repelled flies, 67.8% (80/118) thought they repelled tsetse (65.3%, 62/95 cross-sectional; 78.3%, 18/23 longitudinal) and 4.2% (5/118) did not know of other benefits (3.2%, 3/95 cross-sectional; 8.7%, 2/23 longitudinal). When asked if they considered ticks or tsetse to be more problematic, 40.8% farmers reported ticks (36.1%, 35/97 cross-sectional; 60.9%, 14/23 longitudinal) and 59.2% reported tsetse (63.9%, 62/97 cross-sectional; 39.1%, 9/23 longitudinal).

#### 3.3.7 Risk factors influencing uptake of vector control

**Fig. 5:**
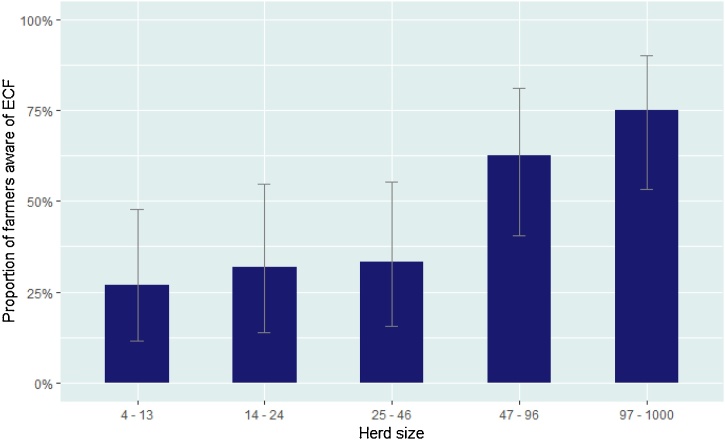
Herd size in relation to farmer awareness of ECF. Error bars indicate 95% confidence intervals.

The relationship between herd size, awareness of ECF and implementation of control measures was also investigated. All questionnaires were combined for this analysis. A significant association was observed for farmers with the largest herds using the spray method (97–1000, OR = 26.6, CI: 1.45–487.91, p = <0.001). The proportion of farmers aware of ECF increased significantly with herd size (47–96 cattle, OR = 4.24, CI: 1.36–14.31, p = 0.013, and 97–1000 cattle, OR = 7.40, CI:2.28−27.09, p = 0.001) (Fig. 5). A significant association between awareness of ECF and the proportion spraying their cattle was observed; the majority of farmers who were aware of ECF were spraying their cattle (89.1%)(OR = 3.19, CI: 1.26–9.07, p = 0.017).

### 3.4 Workshop findings

Focus group discussions allowed for the drivers and barriers of vector control options to be explored (Table 3), as well as raising additional questions for further study. Five methods of control were described for vectors generally or ECF specifically: spraying, dipping, ECF vaccine, bush clearing and avoidance of wildlife and tsetse areas. Only spraying and dipping were considered to be regularly practiced in the area; vaccination was of interest to livestock keepers but not currently conducted. Bush clearing was discussed but is not widely practised. Avoidance of wildlife areas was considered to reduce infection risk and treatment costs, but was seen as challenging due to a lack of grazing in non-protected areas. Livestock-keepers identified cost, time, logistical considerations and safety of cattle and people as the main factors influencing choice of control. Spraying was considered less expensive whilst the more expensive costs of dipping and vaccinating were considered disadvantageous. Spraying was considered to take a long time, compared to dipping being described as time efficient and thus advantageous. An advantage of spraying was that it could be done at home and any time. In contrast, farmers dipping cattle had to follow a set timetable as well as potentially travelling long distances to dip tanks, both considered disadvantages. Farmer safety considerations were discussed, with spraying requiring protective equipment and more potential for farmers to be injured in contrast to dipping. The safety and health of cattle was also a concern related to dipping due to the potential for injury or disease transmission from other cattle.

**Table 3:** Perceived advantages and disadvantages of available control options by livestock-keepers.

Control method	Advantages	Disadvantages
Spraying	Kills ticks and reduces numbers on animals	Does not kill lice
	Simple application	Does not reach the underside of the cattle
	Can use at home, any time	Carrying the sprayer causes pain and is tiring
	Kills ticks in the boma (enclosure) as well	Takes a long time
	Uses small amount of acaricide so reduces cost	Acaricide is wasted
	Do not need an expert to carry out	Requires protective gear, harmful effects of chemicals to farmers
	Cattle do not get wounded	Farmers can be injured by cattle
	Difficult to overdose so does not kill animals	Preparation of acaricide is not correct
	Can use on small numbers of animals	
	Cattle do not get lost	
Dipping	Apply acaricide to many cattle in short time	Meeting other cattle/congestion allows for disease transmission
	Assurance all parasites killed	Expensive
	Acaricide will remain on cattle for 14 days	Lack of education on how to use dip tanks
	Cattle wetted all over body	Cattle can be lost, injured or die during dipping
	Helps to get good quality livestock	Inadequate water sources near dip tanks
	No acaricide wastage	Long distances to travel to tanks (<8km) because not all villages have dip tanks
	Time efficient	Cattle dung reduces efficacy of acaricide
	Prevents tick and tsetse-borne diseases	Set timetable, if miss a date must wait for next
	Safe to farmers, do not need mask	Attendants not managing dip tanks well, profit prioritised over service
	Acaricide prepared properly by livestock professionals	
ECF vaccine	Cattle do not suffer from ECF	Expensive
	Lifelong immunity to ECF	Acaricides still required to prevent other TBDs
		Lack of vaccine availability in this area

## 4 Discussion

The prevalence of *T. parva* in cattle and use of vector and disease control methods were assessed in a wildlife-livestock interface area in Tanzania. Data collected through farmer questionnaires suggest that the low tick burden and associated *T. parva* infection rate is most likely due to the high proportion of farmers using acaricides regularly. However, the application of acaricides was often conducted incorrectly, raising questions about sustainability.

The overall prevalence of *T. parva* was 5.07% (CI: 3.70−7.00%). All cattle sampled were described as healthy at time of sampling and therefore the *T. parva* positive animals are likely to be recovered carrier animals with very low levels of circulating piroplasms, i.e. the cattle-maintained *T. parva* population that is able to differentiate to transmissible infections in cattle. In a separate analysis (Allan et al., 2021), genotyping studies were conducted on the same samples from the study area to assess the genetic diversity of *T. parva* populations circulating in cattle and buffalo, which demonstrated limited sharing of alleles between the two host species, with the alleles mostly being unique to buffalo and a smaller proportion unique to cattle. These data indicate that for the animals sampled and analysed in this study, the *T. parva* is most probably predominantly cattle-derived, although it should be noted that since samples were not conducted from animals suffering from clinical ECF, the degree of impact of buffalo-derived parasites could not be fully assessed. Given the likely carrier status and therefore low parasitaemia of the cattle in this study, 5.07% probably represents an underestimate of the true *T. parva* prevalence. However, this value is still low; other studies that have used the same p104 PCR have reported prevalence of 7.4–60.1% (Laisser et al., 2014; Kazungu et al., 2015a, 2015b; Kerario et al., 2017a). The prevalence was lower than the expected prevalence (20.0%) used to calculate sample size, however the sample size was more than adequate as a smaller prevalence required a smaller sample size (n = 483).

Prevalence did not differ significantly between herds or villages, except for village number 8 with zero prevalence. Interestingly there was no infection detected in village 8 despite close proximity to the wildlife areas. It was not possible to assess seasonality on prevalence as the data were collected over an insufficient time period, however this would be an interesting variable to study.

The odds of *T. parva* infection was 3.47 (CI: 1.39−9.39%) times higher in cattle aged over 4.5 years, compared to those aged between 6 months and 1.5 years. This has been observed in other studies and is likely to indicate cumulative exposure to a sustained tick challenge (Magona et al., 2000; Okiria et al., 2002). Cattle over a year of age in endemic areas tend to be protected from clinical disease, because of acquired immunity after being exposed to infection as calves (Rubaire-Akiiki et al., 2006; Norval et al., 1992).

The survey found very low counts of ticks on cattle, particularly on the ears which is the predilection site for *R. appendiculatus*, where ticks were observed in only 3.7% of cattle. This was surprising; the survey was conducted at the time of year when farmers reported ticks were most commonly found on cattle, and environmental tick distribution studies have reported high abundance of *R. appendiculatus* in the study area (Lynen et al., 2007). Other studies in northern Tanzania have reported high tick burdens and high *T. parva* prevalence in cattle (92% of animals were infested with ticks and *T. parva* prevalence of 38.3% by PCR in Serengeti District (Laisser et al., 2014), and high burdens of >50 per animal and *T. parva* prevalence of 21.8% by PCR in Mara District (Kerario et al., 2017a)). It seems most likely, therefore, that the low counts are due to the vector control that is being implemented by farmers.

The majority of livestock keepers in this area reported frequent use of acaricides. The median frequency of usage was 10 days for spraying and 14 days for dipping. A total of 37.9% of farmers reported spraying weekly, and 4.2% sprayed every four days. All of the products being used advise fortnightly application. Only a small proportion of acaricides used for spraying were being prepared correctly with most being underdosed; even those using an appropriate amount of active ingredient per animal were predominantly using products that were overly concentrated, but in insufficient volumes, consistent with previous studies in Tanzania (Swai et al., 2005, 2009). There is a lack of detailed research about what impact the relative frequency, concentration and dosage are likely to have on efficacy and cost effectiveness, which is perhaps surprising given the extremely widespread usage of these products. Other short or longer term impacts are also unclear. Using overly concentrated products can cause damage to the skin of cattle (Vudriko et al., 2016), is potentially harmful to the farmers and the environment (Swai et al., 2005) and could give rise to toxicity issues in cattle, farmers and the environment (FAO, 1984). Frequent acaricide usage, especially at sub-optimal amounts of active ingredient, raises concerns about sustainability, since it increases the risk of ticks developing drug resistance. Although detailed spatiotemporal data are lacking, resistance of ticks to synthetic pyrethroids and organophosphate acaricides has been described as “widespread” (FAO, 2004) and studies in Uganda using the larval packet test demonstrated high levels of resistance to synthetic pyrethroids in *R. appendiculatus*, in an area where multiple acaricide products were being used (i.e. not solely synthetic pyrethroids) (Vudriko et al., 2016).

All farmers in the study were using a single drug class of acaricide – cypermethrins – without any rotation. It is currently national policy in Tanzania in areas where ticks and tsetse co-occur for formulations that are effective against both vectors to be used (cypermethrins are the preferred class of drug for tsetse (Hargrove et al., 2000)), and government subsidies have been provided for synthetic pyrethroids in such areas (Personal communication, Joyce Daffa, Tsetse Control Division). However, this does raise concerns regarding the potential development of tick resistance to synthetic pyrethroids. Although the study focused on ECF, many farmers reported practicing vector control for both tsetse and ticks; 40.8% farmers in the survey reported ticks to be more problematic and 59.2% reported tsetse suggesting that both are important in farmers’ decisions. Although this study focused on ECF, vector control decisions should be made more holistically. Rotation policies could extend the lifespan for individual acaricides and mixing acaricide classes could delay the development of resistance in ticks (Dolan, 1999). However, this is clearly not straightforward as other acaricide classes are not effective against tsetse, and such a rotation policy would require integration with other tsetse-control methods, in order to maintain the balance between prevention of acaricide resistance in ticks and prevention of disease transmission by tsetse.

The survey highlighted a basic lack of instruction for preparation and application of acaricide for farmers to follow. In some instances the instructions provided did not state the required volume per head or the frequency of application, and often the directions were only provided in English. Although most farmers were familiar with ticks, there was notable variation in awareness of ECF and implementation of control measures. Owners of larger herds were found to be significantly more likely to be aware of ECF and owners who were more aware were more likely to spray their cattle. Although education levels were not measured, it is likely that the farmers of smaller herds in particular may have less access to information, making them more vulnerable. Owners of larger herds are more likely to be engaged with veterinary services, and have better access to veterinary products. There is scope, therefore, to improve efficacy of ECF control by engaging with farmers, particularly those with smaller herds who are less aware of ECF and control options, in order to improve disease awareness and the accuracy of acaricide use. Better labelling of products, including either instructions in Kiswahili or diagrammatic instructions appropriate for low literacy levels, would improve farmers’ ability to accurately prepare acaricides in adequate amounts of active ingredient, which is essential to minimising acaricide resistance in the tick population.

The choices of livestock keepers to dip or spray also depended on the availability of dips in their village; five of the eight villages sampled in the survey had functioning dip tanks. Whilst the Tanzanian government has invested in building and maintaining dip tanks, lack of functional dips is often reported to be a constraint to cattle production (Chenyambuga et al., 2010) and was raised as a concern by livestock-keepers in the workshops. The workshops confirmed that the vector control measures in the study area were predominantly farmer-led, rather than directed by wider vector control strategies.

None of the farmers were using the ITM vaccine. This was predominantly due to lack of awareness of the vaccine, although the cost was also reported to be a concern. The vaccine is used successfully in other parts of Tanzania (Di Giulio et al., 2009; Martins et al., 2010; Lynen et al., 2012) and in other countries in east Africa, including areas where cattle and buffalo interact. A field study in northern Tanzania found no difference in vaccine efficacy whether buffalo were present or not (Homewood et al., 2006), but in contrast, recent field studies in Kenya indicated that the ITM vaccine does not confer protection to cattle in buffalo areas with high tick challenge (Bishop et al., 2015; Sitt et al., 2015). Therefore, the implications for vaccine utility in areas where cattle may become infected with buffalo-derived *T. parva*, such as the study area, remain unclear. *T. parva* genotyping studies in sympatric cattle, buffalo and *R. appendiculatus* populations would be beneficial in order to evaluate the population flow between hosts and vectors, and the potential risk to cattle of buffalo-derived *T. parva*.

The workshops provided useful insights into farmer and veterinary perspectives on vector control. The main drivers for decision-making by farmers were cost, time taken, logistical considerations such as distance to dip tanks or the ability to spray on their own timescale, and safety of both animals and people. This information, which can help to guide development of improved disease control strategies, was not captured in the questionnaire, demonstrating the value of collecting both quantitative and qualitative data. Additional data were collected during group discussions at the workshops, and further qualitative analysis would be valuable to more fully explore drivers and barriers to effective control, in order to optimise disease control in the future. The enthusiasm of all participants – farmers, livestock extension officers and village leaders - to engage with this project demonstrates the importance of ECF control and the potential for collaborative development of future research with the communities involved.

The questionnaire approach used here had some inherent limitations. Although response rates were generally good, some data were not supplied. For example, of the farmers that reported using insecticides, only 68.8% of cross sectional and 85.7% of longitudinal farmers provided sufficient details to calculate dilution of insecticides. The questionnaire enumerator was a local livestock officer known to the farmers so they may have felt pressure to report ‘correct’ answers, although the workhops were used to explore findings in more detail.

Farmers did report cases of ECF and associated mortality, but the nature of this study meant these could not be confirmed. Further studies to quantify true incidence would be valuable. In order to draw more definitive conclusions on the epidemiological state of the study area, baseline data on environmental tick vector numbers and their *T. parva* infection status are required. However, the low prevalence of *T. parva* observed in the survey, the widespread vector control reported by farmers, even if often inadequately administered, and the low numbers of ticks observed on cattle, collectively suggest that significant overt disease is being prevented by application of vector control. It is unclear if cattle in the study area are sufficiently exposed to *T. parva* to result in solid immunity, although the observation in this study of increased prevalence in older animals does suggest ongoing exposure without significant disease. The sustainability of such an acaricide-dependent control strategy, however, remains uncertain - particularly with the use of a single class of acaricide, (Norval, 1981; FAO, 1984; Norval et al., 1985; Torr et al., 2002).

## 5 Conclusions

The study contributes to the understanding of the epidemiology of *T. parva* in Northern Tanzania, and established a low prevalence of *T. parva* infection and low numbers of ticks were found on cattle. Through farmer questionnaires it was evident that the low tick burden and associated *T. parva* infection rate is likely to be due to extensive use of acaricides by the farmers, in particular that of the synthetic pyrethroid cypermethrin. This farmer-led control of ticks and East Coast fever is evidently currently effective, despite farmers often dosing incorrectly. However, the sustainability of such an acaricide-dependent control strategy is questionable, particularly as pyrethroid resistance is known to develop relatively easily in *R. appendiculatus*, even when multiple acaricide compounds are used. While there are government guidelines regarding acaricide use, workshops demonstrated that the vector control in the area was largely farmer-led. Farmers in the study area have good awareness of ticks as an animal health issue, and are using acaricide application throughout the year. Inadequate product instructions likely contribute to the inaccurate dosage use. The extremely widespread use of acaricides highlights the need for further research and education to improve cost-effectiveness. Genotyping studies would be beneficial to understand the flow of parasites between buffalo and cattle, and establish whether use of the ITM vaccine could be a suitable option for an integrated control approach in this area, which would aid farmers in the development of livestock management practices to sustainably control ECF at the wildlife-livestock interface.

## Author contributions

F.K.A., J.S.L., F.M., W.I.M., I.H., L.J.M., H.K.A. contributed to the conception and design of the study. F.K.A., E.S., K.E.A., G.M., E.P., L.J.M., I.H., H.K.A. contributed to the acquisition of data, or analysis and interpretation. F.K.A., E.S., K.E.A., M.B., R.S.L., J.S.L., G.M., E.P., F.M., S.J.T., W.I.M., I.H., L.J.M, H.K.A. contributed to drafting the article or revising it critically for important intellectual content. All authors gave final approval of the version to be submitted.

## Funding

FKA was funded by a KTN/BBSRC iCASE PhD studentship, in partnership with the Global Alliance for Veterinary Medicines (GALVmed) with funding from Bill & Melinda Gates Foundation (Investment ID OPP1093639) and UKAID (Project 300504). S.J.T., L.J.M., H.K.A., J.S.L., R.S.L., F.M. and E.P. were also supported by a grant from the Zoonoses and Emerging Livestock Systems (ZELS) programme (BB/L019035/1). L.J.M., W.I.M., E.P., F.K.A. and the Roslin Institute were supported by a core grant from the UK Biotechnology and Biological Sciences Research Council (BBS/E/D/20231762; BBS/E/D/20002173). The findings and conclusions contained within are those of the authors and do not necessarily reflect positions or policies of the Bill & Melinda Gates Foundation or the UK Government.

## Declaration of Competing Interest

The authors declare that they have no known competing financial interests or personal relationships that could have appeared to influence the work reported in this paper.

## Appendix A Supplementary data

The study questionnaire is presented. Questions about trypanosomiasis were also included since this questionnaire was administered as part of a larger study on vector control for both ticks and tsetse around protected areas.
