# Supplementary material for: A cross-sectional survey to establish Theileria parva prevalence and vector control at the wildlife-livestock interface, Northern Tanzania
Source: Prev Vet Med. 2021 Nov;196:105491. doi: 10.1016/j.prevetmed.2021.105491 (PMC8573586; doi:10.1016/j.prevetmed.2021.105491)
Supplement: Supplementary file 1 [file mmc1.pdf]

Supplementary File: **Questionnaire**

ROSLIN

THE UNIVERSITY of EDINBURGH

Questionnaire for Northern Tanzanian farmers in Serengeti National Park sampling sites

<u>Questionnaire for Farmers in SNP sampling sites</u> Date

Herd/farm identification number

GPS location of farm

Name of participant ……

Head of household? Yes No

If not head of household, please state relationship to head of household ……………………………

……

1.a) How many cattle do you have in your herd **here now**?

b) Do all of the cattle in your herd belong to you? Yes No

c) If they do not all belong to you, how many belong to other people?

d) Are most of your cattle bred on this farm or are most of them bought in?

e) Do you keep your cattle at your farm here overnight? Yes No

If no, where are they kept?……………………………………………………………………………………….......

f) Where do you take your cattle to **graze** and **water**? (Please mark)

	Wet season	Dry season
Where do your cattle **graze**?<br>Location:<br>Time to get there:<br>Distance to get there:		
Do you bring your cattle back to the farm every night? (Yes/No)		
Where do your cattle **water**?<br>Location:<br>Time to get there:<br>Distance to get there:		
Do you bring your cattle back to the farm every night? (Yes/No)		

g) Do you ever send your cattle away for a period of time to a relative/neighbour?

Yes ☐ No ☐

- Do you ever send your cattle away for a period of time to a new area?

Yes ☐ No ☐

If yes, how long for?..

Which months?...

Location (place name):..

How far away (kms)?...

Why?..

h) **How many** other animals are on this farm? Sheep ☐ Goats ☐

2.a) Can you identify a **tick**? (please mark box)

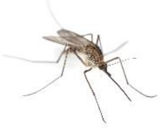	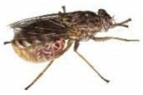	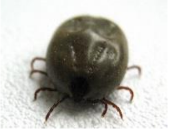	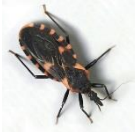	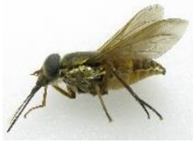

b) Do you see ticks on your cattle? Yes ☐ No ☐

c) On which part(s) of the body do you see ticks?……………………………………………………………………

………

d) In which months do you see ticks? (please circle)

Jan Feb Mar Apr May Jun Jul Aug Sep Oct Nov Dec

e) Which **areas** around your farm (or grazing & watering areas that you use) are ticks most commonly seen?...

………

Around house ☐ When grazing ☐ When watering ☐

When travelling to graze/water ☐ Everywhere ☐

f) Do you see ticks on the ears? Yes ☐ No ☐

g) If yes, which **areas** around your farm (or grazing & watering areas that you use) do cattle pick up ticks **on the ears?**..

………

If yes, what time of year do you see most ticks on the **ears**?..

………

h) Do you know what diseases are spread by ticks?...

………

3.a) Do you do anything to prevent ticks on your cattle? Yes ☐ No ☐

- b) If yes, what do you do? – (please mark as many as apply and mark when used)

	Avoid areas where there are ticks when grazing	Hand removal	Use products to prevent ticks	Use products when you see ticks	Other (please state)
All year round					
Only when ticks are bad (state months)					

- c) If you use products, how do you apply the product(s)? – (please mark box)

Hand spray/pump<br><br>(which part of body)	Brush	Dip tank	Pour-on<br><br>(which part of body)	Other (please state)

<u>If spray/pump</u>

d) What is the time interval between treatments?..

....

e) Product name (if known)..

Take photo of product if available.

f) What dilution do you use the product i.e. product:water ratio?..

....

g) How much of the product do you use in total (for all the cows)? (please indicate if this dose is before or after dilution)..

....

h) Where do you buy the product(s)?..

i) What do the product(s) cost?..

<u>If dip</u>

j) What is the time interval between treatments?...

………

k) Product name (if known)………………………………………………………………………………………………

Take photo of product if available.

l) What dilution do you use the product i.e. product:water ratio?..

………

m) How much of the product do you use in total (for all the cows)? (please indicate if this dose is before or after dilution)...

………

n) Where do you buy the product(s)?...

o) What do the product(s) cost?..

<u>If pour-on</u>

p) What is the time interval between treatments?...

………

q) Product name (if known)………………………………………………………………………………………………

Take photo of product if available.

r) What dilution do you use the product i.e. product:water ratio?..

………

s) How much of the product do you use in total (for all the cows)? (please indicate if this dose is before or after dilution)...

………

t) Where do you buy the product(s)?...

u) What do the product(s) cost?..

v) Does the product just protect against ticks, or does it bring any other benefits?...

………

4.a) Can you identify a **tsetse fly**? (please mark box)

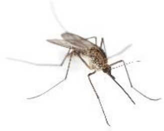	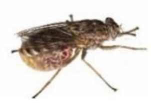	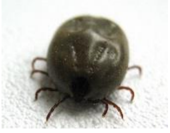	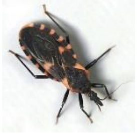	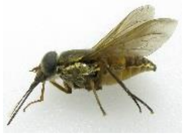

b) Do you see tsetse flies near your cattle? Yes ☐ No ☐

c) In which months do you see tsetse flies?: (please circle)

Jan Feb Mar Apr May Jun Jul Aug Sep Oct Nov Dec

d) Which **areas** around your farm (or grazing & watering areas that you use) are tsetse most commonly seen?………

………

Around house ☐ When grazing ☐ When watering ☐

When travelling to graze/water ☐ Everywhere ☐

e) Do you know what diseases are spread by tsetse?..

………

f) Which cause more problems for your cattle – ticks or tsetse?...

Explain……

………

5.a) Do you do anything to prevent tsetse biting your cattle? Yes ☐ No ☐

- b) If yes, what do you do? – (please mark as many as apply and mark when used)

	Avoid areas where there are tsetse when grazing	Use products to prevent tsetse	Products I use for ticks also protect against tsetse	Other (please state)
All year round				
Only when tsetse are bad (state months)				

- c) If you use products, how do you apply the product(s)? – (please mark box)

Hand spray/pump<br><br>(which part of body)	Brush	Dip tank	Pour-on<br><br>(which part of body)	Other (please state)

<u>If spray/pump</u>

d) What is the time interval between treatments?...

....

e) Product name (if known)...

Take photo of product if available.

f) What dilution do you use the product i.e. product:water ratio?...

g) What dose of product do you use in total (for all the cows)? (please indicate if this dose is before or after dilution)..

h) Where do you buy the product(s)?...

i) What do the product(s) cost?...

<u>If dip</u>

j) What is the time interval between treatments?...

....

k) Product name (if known)...

Take photo of product if available.

l) What dilution do you use the product i.e. product:water ratio?...

m) What dose of product do you use in total (for all the cows)? (please indicate if this dose is before or after dilution)..

n) Where do you buy the product(s)?...

o) What do the product(s) cost?...

If pour-on

p) What is the time interval between treatments?..

....

q) Product name (if known)...

Take photo of product if available.

r) What dilution do you use the product i.e. product:water ratio?...

s) What dose of product do you use in total (for all the cows)? (please indicate if this dose is before or after dilution)..

t) Where do you buy the product(s)?...

u) What do the product(s) cost?...

6.a) Have you heard of East Coast Fever? Yes ☐ No ☐

b) If yes, do you know what the signs of East Coast Fever are? Yes ☐ No ☐

c) If yes, please mark the signs:

Elevated body temperature
Enlarged lymph nodes
Weight loss
Anaemia
Diarrhoea
Rough hair coat
Cough
Others (please state)

d) Do you know what causes East Coast Fever?.................................... Don't know ☐

e) Have any of your cattle had East Coast Fever?

Yes ☐ No ☐ Don't know ☐

f) In the last 1 year,

- How many **cases** of East Coast Fever? ☐
- How many **deaths** from East Coast Fever? ☐

g) Do you have methods to prevent East Coast Fever? Yes ☐ No ☐

h) If yes, please list these methods……………………………………………………………………………………

……

i) Do you use the vaccination to protect against East Coast Fever?

Yes ☐ No ☐

j) If not, why not? (please mark box)

Did not know there was a vaccine	Too expensive	Does not work	Do not know where to buy it	Other (please state)

k) How do you treat cattle you suspect are sick with East Coast Fever? (state preferred treatment)………

……

l) Where do you get treatment from?……………………………………………………………………………

……

7.a) Have you heard of animal Trypanosomiasis? ☐ Yes ☐ No

b) If yes, do you know what the signs of animal Trypanosomiasis are?

Yes ☐ No ☐

c) If yes, please mark the signs:

Elevated body temperature
Enlarged lymph nodes
Weight loss
Anaemia
Diarrhoea
Rough hair coat
Cough
Others (please state)

d) Do you know what causes animal Trypanosomiasis?...

Don't know ☐

e) Have any of your cattle had Trypanosomiasis? Yes ☐ No ☐

f) In the last 1 year,

- How many **cases** of Trypanosomiasis? ☐
- Home many **deaths** from Trypanosomiasis? ☐

g) Do you have methods to prevent animal Trypanosomiasis?

Yes ☐ No ☐

h) If yes, please list these methods ....

....

i) How do you treat cattle you suspect are sick with Trypanosomiasis? (State preferred order)...

....

j) Where do you get treatment from?...

....

Thank you for taking the time to complete this questionnaire, your time and input is greatly appreciated and the valuable information you have given will help to better understand the diseases mentioned.
